# Deep Learning and Transfer Learning for Malaria Detection

**DOI:** 10.1155/2022/2221728

**Published:** 2022-06-29

**Authors:** Tayyaba Jameela, Kavitha Athota, Ninni Singh, Vinit Kumar Gunjan, Sayan Kahali

**Affiliations:** ^1^Department of Computer Science & Engineering, JNTUH College of Engineering, Hyderabad, India; ^2^Department of Computer Science and engineering, CMR Institute of Technology Hyderabad, India; ^3^Department of Computer Science and Engineering, University of Science and Technology Chittagong, Chittagong, Bangladesh; ^4^Department of Radiology, Washington University, Saint Louis, USA

## Abstract

Infectious disease malaria is a devastating infectious disease that claims the lives of more than 500,000 people worldwide every year. Most of these deaths occur as a result of a delayed or incorrect diagnosis. At the moment, the manual microscope is considered to be the most effective equipment for diagnosing malaria. It is, on the other hand, time-consuming and prone to human error. Because it is such a serious global health issue, it is important that the evaluation process be automated. The objective of this article is to advocate for the automation of the diagnosis process in order to eliminate the need for human intervention in the process. Convolutional neural networks (CNNs) and other deep-learning technologies, such as image processing, are being utilized to evaluate parasitemia in microscopic blood slides in order to enhance diagnostic accuracy. The approach is based on the intensity characteristics of *Plasmodium* parasites and erythrocytes, which are both known to be variable. Images of infected and noninfected erythrocytes are gathered and fed into the CNN models ResNet50, ResNet34, VGG-16, and VGG-19, which are all trained on the same dataset. The techniques of transfer learning and fine-tuning are employed, and the outcomes are contrasted. The VGG-19 model obtained the best overall performance given the parameters and dataset that were evaluated.

## 1. Introduction

Malaria is spread through the bites of female *Anopheles* mosquitoes infected with *Plasmodium* protozoan parasites, which infect red blood cells and cause them to swell and swell up. Every year, 3.2 billion people worldwide are at high risk of developing malaria, according to the World Health Organization. According to a survey conducted by the World Health Organization [1], 91 countries recorded 216 million cases of malaria. The World Health Organization is a nongovernmental organization that promotes health worldwide. Global malaria cases were primarily concentrated in the African Region, which was then followed by the Southeast Asia Region and the Eastern Mediterranean Region. The symptoms of malaria are often associated with fever, tiredness, headaches, and, in extreme cases, seizures and coma, all of which can be fatal if not treated promptly. Malaria is a preventable disease that can be controlled with adequate treatment. There is, however, no effective immunization available at this time. Once infected, it is a disease that progresses at a rapid pace. Malaria is a significant load on our healthcare system, and it is the top cause of death in many developing and developing-country populations. It is endemic in many parts of the world, which means that the disease is met on a regular basis in those areas of the world. As a result, early detection and treatment of malaria are essential in order to save lives. Because of this, we are motivated to increase the effectiveness and timeliness of malaria diagnostics in the future. Specialized technology is required in order to resolve this problem. As a result, it is vital to obtain a prompt diagnosis. The most important task in diagnosing malaria is to determine whether or not parasites are present. The most common method of diagnosing malaria is by the use of a blood sample. In the United States alone, millions of blood samples are tested for malaria each year, with a trained pathologist painstakingly counting parasites and infected red blood cells in each sample. According to the World Health Organization regulation [2], the blood smear should be inspected under a microscope at a magnification of 100x. Diagnostic treatments such as light microscopy and rapid diagnostic tests are two of the most often performed (RDT). The use of these two tests is typical in situations where high-quality microscopy services are not readily available. However, there are several disadvantages to using these procedures, including the fact that the diagnosis is primarily dependent on the pathologist's knowledge and skill, the possibility of false-positive and false-negative diagnoses, which can result in the development of other illnesses, and the fact that they are time-consuming, to name a few.

Late or incorrect diagnosis is the leading cause of death in the United States. As a result of the severity of this global health concern, it is important that the evaluation process be automated. The proposed approach must be capable of identifying parasitemia while also providing a more trustworthy and consistent interpretation of blood films, among other things. It must be cost-effective and alleviate the load placed on malaria field workers and their families.

In today's world, deep learning algorithms are commonly used to classify photos, recognize films, and analyze medical images, among other things. Convolutional neural networks (CNNs), a kind of deep neural networks, are the neural networks that are most commonly utilized in the field of computer vision. Specifically, in the field of biomedicine, deep neural networks have been demonstrated to be the most effective machine learning technology available. Due to the ease of extraction of crucial information and completion of tasks that were previously difficult to complete using conventional approaches, deep learning (DL) has become highly popular in the recent decade for evaluating and diagnosing biomedical and healthcare problems. The convolutional layer of the CNN serves as an automatic feature extractor, extracting both hidden and important properties from the input data. Image categorization is accomplished by the use of a fully connected neural network, which optimizes probability scores by feeding the retrieved features into the network. Additionally, when deep learning is used in biological applications, the number of research articles published has increased significantly over the past several years. There are three broad categories of applications for machine learning in biomedical applications: (1) as a computer-aided diagnosis to assist physicians in making more accurate and timely diagnoses, with improved harmonization and fewer contradictory diagnoses; (2) to improve patient medical care through more personalized therapies; and (3) to improve human wellbeing, for example, through the analysis of disease spread and social behavior in relation to environmental factors [3]. Medical devices and equipment are now capable of producing vast amounts of data, which can include photos, audio, text, graphs, and signals, among other types of information. Using a machine learning technology known as deep learning, this medical data may be analyzed [4]. Deep learning is a technique that consists of layers of comparable functions cascading down through the network. Deep-learning algorithms can mine massive amounts of healthcare data in search of information that can be used to aid in the treatment and prevention of diseases and ailments. Deep-learning algorithms can mine massive amounts of healthcare data in search of information that can be used to aid in the treatment and prevention of diseases and ailments. People who are knowledgeable in the machine learning area recognize the global impact that deep learning is having by investigating and resolving human problems across all fields, despite the fact that deep-learning applications may appear disillusioning to the general individual.

A fatal disease, malaria affects hundreds of millions of people each year all over the world, and it is preventable. If it is not treated immediately, it can be fatal. Although there have been significant advancements in malaria diagnosis, the microscopy approach continues to be the most extensively employed. Unfortunately, the accuracy of microscopic diagnostics is dependent on the expertise of the microscopist, resulting in a limitation in the throughput of malaria diagnosis. Manual microscopy has been proved to be an unreliable screening method when conducted by nonexperts due to a lack of training, which has been demonstrated in several investigations, particularly in rural areas where malaria is endemic. An automated system's mission is to do this activity without the need for human interaction, and it should do so by providing a goal-oriented, dependable, and efficient tool to accomplish this. It is now possible to minimize expenses while simultaneously enhancing overall accuracy because of the advent of artificial intelligence tools, notably deep-learning techniques. In this study, we present a VGG-based model for recognizing infected cells, and we compare it to previously created models in order to demonstrate its effectiveness. Our model outperforms the majority of previously produced models over a wide range of accuracy metrics. The model has the advantage of having a modest number of layers because it was constructed in this manner. Thus, the number of computing resources and computational time required are kept to a minimum.

## 2. Related Work

Plasmodium parasites transmit malaria, a disease that can be fatal if untreated. Specialized microscopists use specialized equipment to look for it in very small blood smear images. It is possible that modern deep-learning algorithms may be used to accomplish this study on a computer. One of the most notable results of using deep learning in the medical industry is that it can recognize malaria, which is in line with earlier findings, as demonstrated by this study. These findings have been put to use in the creation of a new deep learning-based system for diagnosing malaria illness. If an autonomous model can be developed that is exact and effective, it can reduce dramatically the demand for highly qualified employees. Here, we present the Malaria Diagnosis System, a fully automated convolutional neural network- (CNN-) based model for the detection of malaria in microscopic blood smears (MDS). Computing algorithms have been used extensively over the past few decades to develop cost-effective healthcare solutions in the context of chronic sickness reduction. The development of several artificial intelligence technologies has made it possible to diagnose malaria using blood smear images that have gotten increasingly complex in recent years. Convolutional neural networks and support vector machines are just some of the techniques that can be used in artificial neural networks (ANNs) and convolutional neural networks (CNNs) (SVMs). Deep learning is the most recent innovation that has had a positive impact on a wide range of industries, including but not limited to medicine. This advanced version of the well-known multilayer neural network automatically learns complicated data representations (also known as features) from large amounts of data in a short period of time. A vast collection of high-quality, annotated data is required for deep-learning models to learn and generate accurate predictions about future occurrences, but a little amount of data is sufficient for machine learning algorithms. Because it is more difficult to collect annotated training sets and because many privacy issues occur, it is possible that this is one of the reasons why the medical domain was unable to accept the new technology during its early phases of development. Unexpectedly, trained deep-learning models can be used to solve problems in a variety of different but related applications through the use of a technique called transfer learning, which is described here. These trained deep-learning models are also referred to as pretrained models, which are models that have already learned to deal with an issue that is similar to the one that is now being addressed. Transfer learning is one of three ways for incorporating deep-learning algorithms into a training environment. The availability of a large amount of labeled data adds to the promise of CNNs' performance in a variety of applications. DL approaches are currently being used by researchers all around the world to yield promising outcomes in a variety of medical image analysis and interpretation applications [5]. When it comes to breast cancer diagnosis, Zhang and colleagues [6] proposed a nine-layer convolutional neural network that had a 94 percent accuracy rate [7]. Attempts of a similar kind have been made to identify tuberculosis disease, with higher performance accuracy being seen. Using a customized convolutional neural network- (CNN-) based deep-learning (DL) model, Sivaramakrishnan et al. and colleagues [8] investigated the visualization of salient network activation in a job of chest X-ray screening using a deep-learning (DL) model. A new object detection technique developed by Jane Hung and Anne Carpenter [9] is based on a Faster Region-based convolutional neural network (Faster R-CNN) that was trained on ImageNet before being fine-tuned on their dataset. The Faster R-CNN was trained on ImageNet before being fine-tuned on their dataset. On ImageNet, the Faster R-CNN was trained and fine-tuned until it performed well.

Other studies on the use of DL methods to the challenge of detecting malaria parasites have been published in the literature as well.

A designed dataset of 2,565 cell photos was used by Dong et al. [10] to investigate the performance of SVM with pretrained DL models such as LeNet [11], AlexNet [12], and GoogleNet [13] on a tailored dataset of parasitized and uninfected cells in discriminating between them. The authors divided the red blood cells (RBCs) from thin blood smear images into two groups: train sets and test sets, using a random number generator. To validate the models, a total of 25% of the training photographs were chosen at random from the pool of photos. In order to accommodate a large number of whole slide photos in the dataset, the image size submitted to LeNet and AlexNet was 60 × 60; however, GoogleNet accepted an image size of 256 × 256. Aside from outperforming SVM on their unique dataset, the pretrained models outperformed each other, with GoogleNet providing the highest accuracy (98.13 percent) out of all the pretrained models they examined.

For distinguishing between parasitized and uninfected cells, Liang et al. [14] proposed a 16-layer CNN as a possible solution. Using the pretrained AlexNet as a basis for feature extraction, and using the extracted features as the basis for training an SVM classifier, the performance of their proposed model was compared to that of a CNN that had already been trained. It was determined by the researchers' findings that the customized model surpassed the pretrained model in terms of accuracy as well as sensitivity and specificity.

Bibin et al. [15] suggested a 6-layer deep belief network for detecting malaria parasites in peripheral blood smear images, which they found to be effective. The researchers used randomized train/test splits to obtain 96.4 percent accuracy in categorizing their customized dataset of 4,100 cells, according to their findings.

When asked to discriminate between parasitized and uninfected cells in an image dataset of 27,558 cell photos, Shaik et al. [16] proposed a customized, sequential CNN with three convolutional layers and two fully connected layers, which they found to be effective. Using pretrained CNNs, such as AlexNet, VGG-16 [17], Xception [18], ResNet-50 [19], and DenseNet-121 [20], the authors evaluated the effectiveness of the CNNs in extracting attributes from parasitized and uninfected cells. For the AlexNet and VGG-16 models, features were extracted from the second fully connected layer, while for the Xception, ResNet-50, and DenseNet-121 models, features were extracted from the last layer before the final classification layer. With an accuracy of 95.7 percent, ResNet-50 surpassed the other pretrained CNNs and customized CNN models in every performance criterion, according to the researchers.

With the help of transfer learning and fine-tuning, Prasad et al. [21] compared the performance of a pretrained model VGG-16 with transfer learning and fine-tuning to a 19-layer custom architecture with eight convolution layers, four max pool layers, three dense layers, one flattens layer, two layers with 50% dropout (to reduce overfitting), and one fully connected layer. The results revealed that VGG-16 performed the best, achieving an accuracy rate of 97.77 percent. By contrast, using the dropout technique, there is an increased risk of losing information from the image [22].

AOCT-NET [23] is an 18-layer transfer learning architecture developed by Suryanarayana et al. [24]. The authors of this paper compared the performance metrics of AOCT-NET to those of contemporary architectures in the literature. In terms of evaluation, the former obtained the greatest possible score.

After applying transfer learning, Alqudah [25] investigated the performance of a custom CNN architecture as well as the pretrained models VGG-16 and VGG-19 [17] and discovered that VGG-19 outperformed the other models in their study (95.33 percent). With the inclusion of new optimization methods, this accuracy has the potential to improve.

A deep-learning library that provides practitioners with high-level components that can rapidly and readily deliver results in traditional deep-learning domains and low-level components that can be combined to construct new techniques [26] was used in this study. In order to achieve these goals, it does not intend to make substantial concessions in terms of usability, flexibility, or performance. It is based on PyTorch [27], which gives the neural network a slew of new features, such as data visualization tools, new ways to import and partition data, and the ability to infer the dataset's class count. PyTorch is used to build Singh and Ahuja [28]. Deep-learning algorithms of the present day may be able to automate the process of carrying out this analysis. The development of an autonomous, precise, and efficient model has the potential to significantly reduce the requirement for highly qualified workers. In light of the difficulties associated with manual diagnosis, it is recommended that the malaria diagnosis technique be automated. The automation of the diagnosis process will result in more accurate disease diagnosis and, as a result, has the potential to offer trustworthy healthcare to areas with low resource availability. Because of this, computerized diagnostics may be of significant help to remote areas that lack specialized infrastructure and skilled employees in the first place. Adapting standard microscopy processes, experience, practices, and information to a computerized system architecture is required in order to automate the malaria diagnosis process. Here, we present the Malaria Diagnosis System, a fully automated convolutional neural network- (CNN-) based model for the detection of malaria in microscopic blood smears (MDS). We demonstrated the efficiency of our deep-learning-based method by detecting malarial parasites from microscopic pictures with 97.2 percent accuracy.

## 3. Process Flow and Algorithm

**Figure 1:**
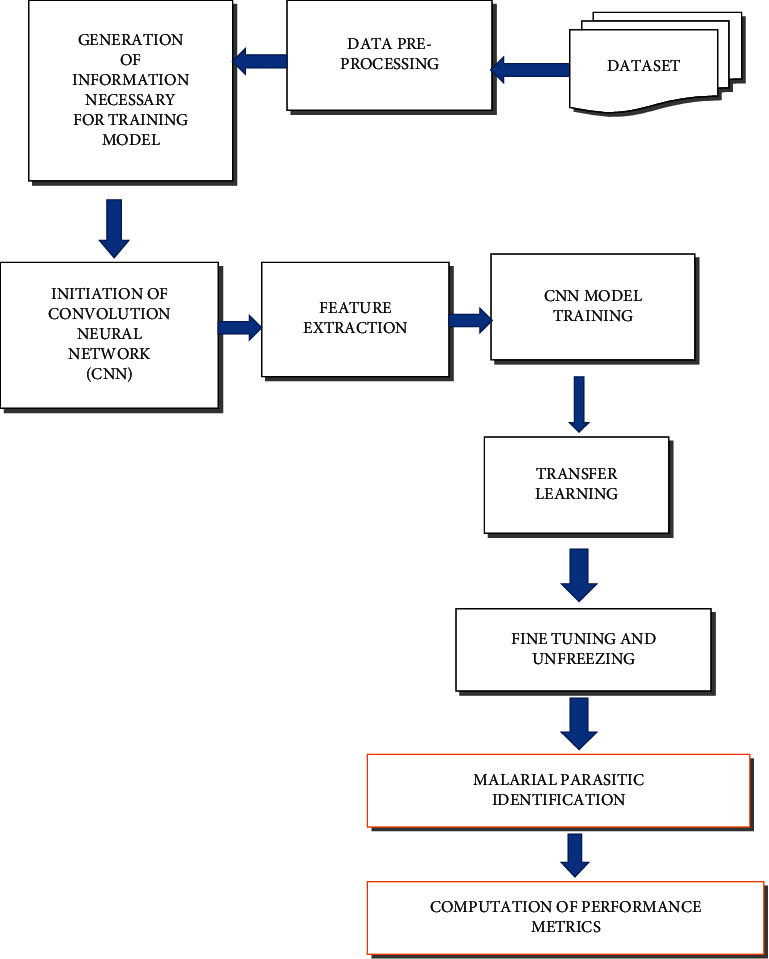
Process flow diagram of the proposed methodology.

The detection of malaria using deep learning is visualized by means of a process flow diagram as shown in Figure 1. It depicts the step-by-step procedural flow of the processes involved in the entire work in an informal illustration.

### 3.1. Data Preprocessing

A model's behavior and performance are completely dependent on the data that it receives when learning is performed through supervised learning. Experiments would be impossible to conduct without the use of data preprocessing. Data Augmentation is used to resize or normalize images before they are fed into the “Learner” class, which collects all of the information required to train a model based on the data. Fastai performs Data Augmentation to resize or normalize the input images before feeding them into the “Learner” class.

### 3.2. Convolutional Neural Network (CNN)

The convolutional neural network (CNN) is one of the deep neural networks that are most extensively utilized today (CNN). As a result of the convolution process, it is named after the linear mathematical action between matrices that is used to create it [29]. The architecture of CNN is comprised of four layers: a convolutional layer, a nonlinearity layer, a pooling layer, and a fully connected layer. For the nonlinearity and pooling layers, there are no settings available; however, there are options for the convolutional and fully connected layers. When compared to standard neural networks, CNNs are capable of preserving the spatial correlations of the input while extracting feature information. Weights and biases can be taught for each neuron in a layer by experimenting with them. Data can be fed into the network, and the loss function at the top layer can be minimized to achieve the optimal model. A variety of CNN designs have been proposed, each with its own advantages and disadvantages. In this work, the ResNet-50, VGG-16, and ResNet-19 CNN models were all tested on the same dataset, and the results were compared.

### 3.3. CNN Model Training

The dataset contains both training and validation sets, which are complementary. Approximately 80 percent of the training set is used for real training, with the remaining 20 percent being used for back-propagation validation throughout model training as mentioned in Table 1.

The performance evaluation criteria for this study are accuracy, sensitivity, specificity, precision, and the *F*1 score, among other things.

### 3.4. Transfer Learning

It is the transfer of knowledge from a previously mastered task that improves learning in a new activity [30]. Transfer learning is a machine learning research subject that relies on retaining acquired knowledge while solving one issue and applying it to another but similar problem. Starting with a pretrained model, we change it to predict the two categories of blood smeared photographs using our dataset instead of predicting thousands of categories of ImageNet using the ImageNet dataset.

The final piece of the model has to be reworked to fit our amount of classes in order to work. A few linear layers are commonly seen toward the end of most convolutional models (a part we will call the head). Convolutional neural networks can identify and analyze features in an image as it travels through convolutional layers. The head's role here is to translate these data into predictions for each of our classes. A new head with a random initialization technique will be built during transfer learning, preserving all of the convolutional layers (also known as the model's backbone) and their weights that have been pretrained on ImageNet.

Next, we will unfreeze the layers of the backbone and fine-tune the entire model. First, we will freeze the body weights and train only the head (in order to turn the assessed characteristics into predictions for our own data) (possibly using differential learning rates).

### 3.5. Fine-Tuning and Unfreezing

Fine-tuning [31–35] consists in removing the final set of fully linked layers from a pretrained CNN and replacing them with a new set of fully linked layers. All of the layers below the head are frozen, and their weights cannot be changed because of this. Unfreeze allows us to choose which layers of your model to train at any given time by removing them from the freeze state. This is due to the fact that the early layers of our model will already be well trained in recognizing basic lines, patterns, and gradients, whereas the later layers (which will be more specific to our aim, such as identifying parasitemia) will necessitate further training [36–39]. By fine-tuning pretrained networks, we may utilize them to recognize classes that they were not trained on in the first place. Furthermore, this method has the potential to be more accurate than feature extraction-based transfer learning in terms of accuracy and precision (Algorithm 1).

## 4. Dataset Description

### 4.1. The Dataset

**Figure 2:**
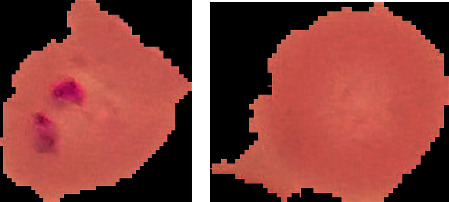
Sample images of parasitized (a) and uninfected (b) red blood cells (RBCs).

The researchers used a dataset of 27,558 segmented red blood cells (RBCs) with an equal proportion of parasitized and uninfected cells in order to conduct their investigation. Blood smear images of healthy and sick blood smears have been painstakingly collated and analyzed by researchers at the Lister Hill National Center for Biomedical Communications (LHNCBC) of the National Library of Medicine. It is possible to see in Figure 2 the difference between malaria-affected and unaffected red blood cells (RBCs) based on data collected from the dataset.

Simonyan and Zisserman [17] of Oxford University created a conventional multilayered convolutional neural network (CNN) architecture, known as the Visual Geometry Group (VGG). The VGG achieved remarkable results for the ImageNet Challenge. This design serves as the foundation for cutting-edge object recognition models, which are built on the VGG architecture. On a range of tasks and datasets other than ImageNet, the VGGNet, which was developed as a deep neural network, exceeds the baselines in terms of performance. Besides that, it is still one of the most extensively used image recognition architectures on the market today. The two most popular models are VGG16 and VGG19, which have 16 and 19 convolutional layers, respectively, and are the most widely used [40–42].

**Figure 3:**
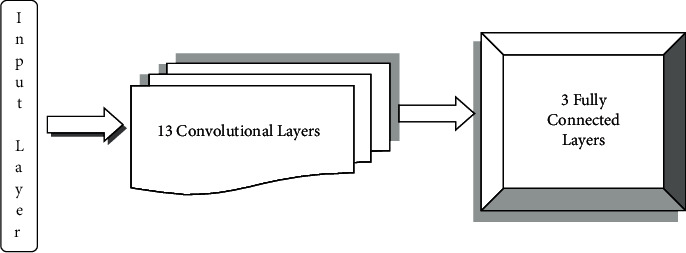
VGG-16 architecture.

The VGG-16 consists of 13 convolutional layers and three fully connected layers. In ImageNet, the VGG16 model achieves nearly 92.7% top-5 test accuracy [43].  Figure 3 shows the VGG-16 architecture. Following a few convolution layers, there is a pooling layer that reduces the height and width. When it comes to the number of filters that can be used, there are approximately 64 available, which can be doubled to approximately 128 and then to 256 filters. We can use 512 filters in the final layers. The model has an image input size of 224 × 224.

When it came to classification and localization at the 2014 ILSVRC conference in Chicago, the VGG-19 model, with a total of 138M parameters, was ranked second. In the ImageNet Large-Scale Visual Recognition Challenge, this model was trained on a portion of the ImageNet database, which was then used to compete in the competition (ILSVRC).

Due to the fact that the input image was a rectangle RGB image with a fixed size (224*∗*224), a rectangular matrix was used in this network. The photos were first submitted to a single preprocessing step, which consisted in eliminating the average RGB value from each pixel, which was computed throughout the whole training set and employing kernels with a stride size of one pixel. Consequently, they were able to express the full significance of the photograph. Spatial padding was used to ensure that the spatial resolution of the image was not compromised. Stride 2 was used to get the maximum pooling, with a 2 × 2 pixel window for the window size. This was followed by the Rectified linear unit (ReLu), which included nonlinearity in the model in order to improve classification and minimize processing time, whereas earlier models relied on tanh or sigmoid functions to achieve these goals, respectively. This proved to be significantly superior to the other options available. ILSVRC classification was performed using three completely linked layers, the first two of which were 4096 channels in size, the third of which was 1000 channels in size for 1000-way ILSVRC classification, and the last layer was a softmax function.

ResNet is an abbreviation for residual network, which is what it stands for. The theory of deep residual learning for image recognition was first proposed by He et al. in their article titled 'Deep Residual Learning for Image Recognition' [19] in 2015. In the ILSVRC 2015 classification competition, this model was a resounding success, with an error rate of only 3.57 percent, as evidenced by the fact that its ensemble was awarded first place in the classification competition. Additionally, it took first place in the 2015 ILSVRC & COCO contests in a variety of categories, including ImageNet detection, ImageNet localization, coco detection, and coco segmentation. Deep residual nets make use of residual blocks to improve the accuracy of the model. Specifically, the ResNet-34 and ResNet-50 variants of the ResNet network were used in this investigation.

This architecture, known as ResNet-34, employed shortcut connections to transform a plain network into its residual network counterpart. It was the first ResNet architecture. While the simple network was influenced by VGG neural networks (VGG-16 and VGG-19), the convolutional networks were influenced by 33 filters, which was the case in this instance. On the other hand, ResNets are less complex than VGGNets and feature fewer filters than these neural networks. The performance of the 34-layer ResNet is 3.6 billion FLOPs, whereas the performance of smaller 18-layer ResNets is 1.8 billion FLOPs, according to the researchers. The algorithm also adhered to two simple design principles: each layer had the same number of filters for the same output feature map size, and if the output feature map size was cut in half, the number of filters was doubled in order to maintain the time complexity of each layer. Shortcuts are now available on this straightforward network. Despite the fact that the input and output dimensions were both the same, the identity shortcuts were used directly. The proportions of the space became increasingly huge, and there were two possibilities to choose from. The first was that the shortcut would continue to conduct identity mapping while padding extra zero entries to increase the dimension of the data set being processed. Using the projection shortcut, it was also possible to match up dimensions.

A fundamental adjustment has been made to the ResNet-34 paradigm in order to create the ResNet-50 architecture. Because of concerns about the amount of time necessary to train the layers, the building block was turned into a bottleneck design in this situation. This time, a three-layer stack was used instead of the two-layer stack that was previously used. It was as a result that each of the two-layer blocks in the Resnet34 architecture was replaced by a three-layer bottleneck block, creating the Resnet50 architecture. Accuracy is improved as compared to the 34-layer ResNet model by using this model. In terms of performance, ResNet's 50-layer performance is 3.8 billion FLOPS.

## 5. Results and Analysis

After performing Data Augmentation, the pretrained CNN models were fitted with the dataset to perform transfer learning. The layers were frozen, and fine-tuning was applied. Accuracy results before and after applying fine-tuning have been recorded. The confusion matrix for each of these models has been plotted to evaluate the performance metrics.

### 5.1. Model Performance before and after Fine-Tuning

The performance of the models after fine-tuning was observed to be better than with transfer learning alone.  Table 2 shows the results of accuracy obtained for the transfer learning models before and after fine-tuning.

**Figure 4:**
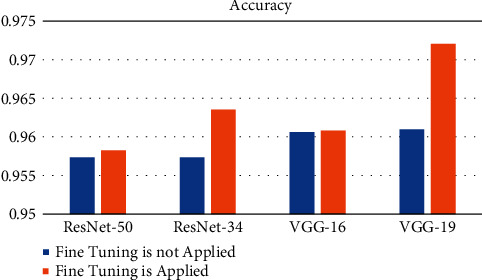
Accuracy representation before and after fine-tuning.

According to Figure 4, the accuracy of the transfer learning models before and after fine-tuning is the same in graphical form for both cases. We can employ pretrained networks to recognize classes that they were not initially programmed to recognize by fine-tuning their responses to them. A lower level of accuracy can be achieved using transfer learning via feature extraction, on the other hand.

### 5.2. Performance Metrics

Performance metrics are used to evaluate the model's overall performance. When determining their worth, a confusion matrix is employed to determine their worth. In machine learning classification problems involving two or more alternative outputs, a confusion matrix can be used to evaluate the problem. In Table 3, there are four different combinations of anticipated and actual data to consider. For the sake of comparison, the matrix of confusion is displayed in relation to the validation dataset.

The accuracy of a prediction is defined as the proportion of correctly predicted observations to the total number of observations. A good measure of accuracy is only possible when we have symmetric datasets with about equal numbers of false positives and false negatives. The default accuracy measure gives an overall statistic for model performance throughout the entire dataset, and it is used in conjunction with other metrics. However, when the distribution of classes is unequal, overall accuracy may be deceiving, and it is critical to correctly predict the minority class in order to avoid bias.

(1) $$\begin{array}{c} \text{Accuracy}=\left(\text{TP}+\text{TN}\right)/\left(\text{TP}+\text{FP}+\text{FN}+\text{TN}\right). \end{array}$$

As a result, more parameters must be incorporated into our model's performance evaluation in order to be accurate. Accuracy is essentially a measure of how frequently the classifier makes an accurate guess. The accuracy of a forecast is defined as the ratio of the number of correct forecasts to the total number of predicted events.

The presence of positive samples that are accurately classified in relation to the total number of correctly classified positive samples is defined as precision in statistics (either correctly or incorrectly). To put it another way, accuracy refers to a model's capacity to correctly detect whether or not a sample is positive. The number of expected good outcomes divided by the total number of predicted outcomes can be used to calculate precision if you want to know how accurate your predictions are.

(2) $$\begin{array}{c} \text{Precision}=\text{TP}/\left(\text{TP}+\text{FP}\right). \end{array}$$

The sensitivity of a class is defined as the proportion of precisely predicted positive observations to all of the observations in the class. The capacity of a model to predict true positives in each accessible category is measured by its sensitivity, which is a numerical statistic.

(3) $$\begin{array}{c} \text{Sensitivity}=\text{TP}/\left(\text{TP}+\text{FN}\right). \end{array}$$

Specificity is defined as the proportion of accurately predicted negative observations to all other observations in the class, divided by the total number of observations. Specificity is a metric that is used to evaluate a model's ability to predict true negatives in each of the categories that are available. This means that any category model can be evaluated using sensitivity and specificity measurements.

(4) $$\begin{array}{c} \text{Specificity}=\text{TN}/\left(\text{TN}+\text{FP}\right). \end{array}$$

In order to calculate the *F*1 score, precision and sensitivity are combined and weighted together. False positives and false negatives are taken into account while computing this score, which is why it is so accurate. While *F*1 is less intuitive than accuracy, it is often more beneficial than accuracy, especially when the distribution of the class is asymmetrical, as seen in the following example. Precision is the most efficient strategy when the costs of false positives and false negatives are equal. Precision and sensitivity should be taken into account because the cost of false positives and false negatives may be dramatically different. Precision and recall measurements are discussed in detail in this section. When precision matches recall, the effect is the greatest.

(5) $$\begin{array}{c} F1\text{ score}=2*\left(\text{sensitivity}*\text{precision}\right)/\left(\text{sensitivity}+\text{precision}\right). \end{array}$$

The number of digits in percentage terms used to display a value is referred to as precision (PR), whereas recall assesses completeness by calculating what percentage of positive data is labeled as such, and the harmonic mean of recall and precision provides an *F*-score that falls between [0, 1].

### 5.3. Classification Report

Based on the results provided in Table 4, the models were successful in distinguishing between infected and noninfected cells.

**Figure 5:**
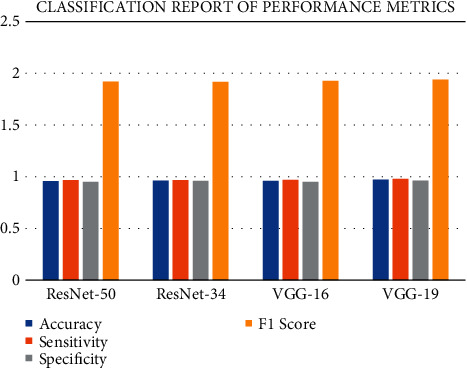
Classification report of performance metrics.

Figure 5 illustrates the classification report for the performance metrics of the dataset that was employed. In order to determine the accuracy of a classification algorithm's predictions, a classification report must be generated for each classification algorithm. Count the number of correct predictions versus the number of wrong predictions. Furthermore, the metrics of a classification report are projected based on the number of true positives, false positives, and true negatives that occur.

The results show that VGG-19 has a superior performance compared to the other pretrained models. An accuracy of 0.972055, sensitivity of 0.979671, specificity of 0.964166, and *F*1 score of 1.939950 have been achieved.

Accuracy is a statistic that can be used to assess the effectiveness of categorization techniques. Our model's accuracy is defined as the percentage of true predictions made by our model in a general sense. In computing, a true positive or true negative value is a data item that was accurately classified as true or false by the algorithm. A false positive or false negative, on the other hand, refers to a data item that was incorrectly classified by the algorithm and so classified as such. A different name for sensitivity is the True Positive Rate (TPR). It is also referred to as recall in some circles. Essentially, it informs us of the proportion of true positive cases that our model predicted to be positive in the beginning. The presence of an extremely high sensitivity score suggests that our model has a great degree of success in correctly predicting actual positives. When an observation does not belong to a predetermined category, specificity has an impact on a model's ability to estimate the future. When an observation actually belongs to a category different than the one under investigation, it is necessary to have an understanding of the model's performance. When the model makes a large number of incorrect positive classifications or a small number of correct positive classifications, the denominator increases, and the precision declines.

**Figure 6:**
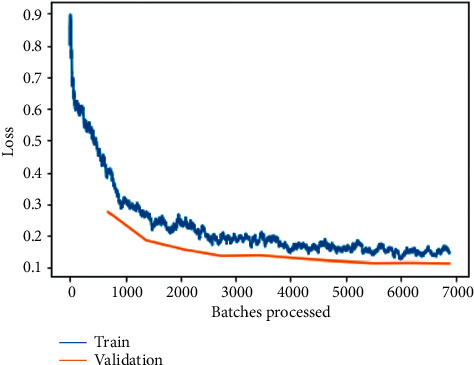
Training and validation loss on the basic ResNet-50 model.

As depicted in Figure 6, the training and validation loss before fine-tuning is plotted between loss and the number of batches processed for the ResNet-50 model training and validation loss is one of the most commonly utilized metrics combinations. The plot of training loss diminishes with experience, whereas the plot of validation loss decreases to a point and then begins to increase again. The training loss will tell us whether or not our model can fit the training set at all, or if our model has enough ability to analyze the important information in the data. The training loss of the model demonstrates how well it fits current data, whereas the validation loss exposes how well it fits new data as input. The major goal is to see both the training and validation losses decrease. While both losses should ideally be nearly the same, as long as the validation loss remains reasonably close to the training loss. A deep-learning model's fit to training data is measured using the training loss. However, validation loss is used to evaluate the performance of a deep-learning model in the validation set.

**Figure 7:**
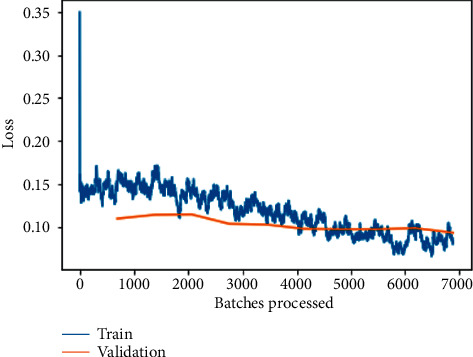
Training and validation loss on the fine-tuned ResNet-50 model.

Loss can be seen to decrease exponentially as the number of batches being processed increases, as seen in Figure 7 for the fine-tuned ResNet-50 model training and validation loss, but with a slight tilt of obtaining constant lowered values, indicating an approximately straight line, as shown in Figure 6. Both scenarios have a lower validation loss because training loss is assessed during each epoch, whereas validation loss is calculated after each epoch in the first instance.

**Figure 8:**
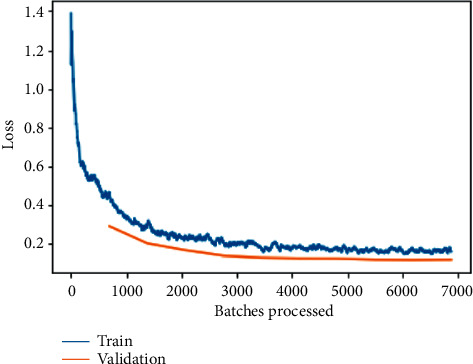
Training and validation loss on the basic ResNet-34 model.

The training and validation loss before fine-tuning as shown in Figure 8 is a plot between loss and the number of batches processed for the ResNet-34 model. One of the most widely used metrics combinations is training and validation loss. The training loss is a metric used to assess how a deep-learning model fits the training data. That is to say, it assesses the error of the model on the training set. Note that the training set is a portion of a dataset used to initially train the model. Computationally, the training loss is calculated by taking the sum of errors for each example in the training set. It is also important to note that the training loss is measured after each batch. This is usually visualized by plotting a curve of the training loss. On the contrary, validation loss is a metric used to assess the performance of a deep-learning model on the validation set. The validation set is a portion of the dataset set aside to validate the performance of the model. The validation loss is similar to the training loss and is calculated from a sum of the errors for each example in the validation set.

**Figure 9:**
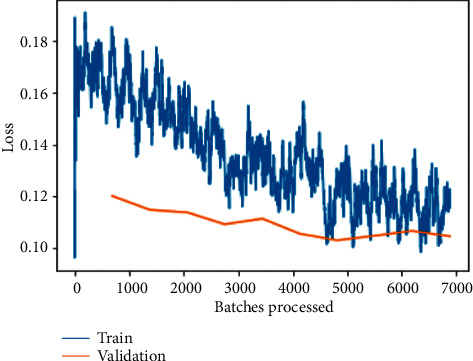
Training and validation loss on the fine-tuned ResNet-34 model.

Here, it can be observed that loss is substantially being reduced exponentially as the number of batches is increased for processing; the same can be observed for training and validation loss on the fine-tuned ResNet-34 model as shown in Figure 9 but with a little tilt of attaining constantly reduced values indicating an approximate straight line. In both the conditions, validation loss is less when compared to training loss because training loss is measured during each epoch while validation loss is measured after each epoch.

**Figure 10:**
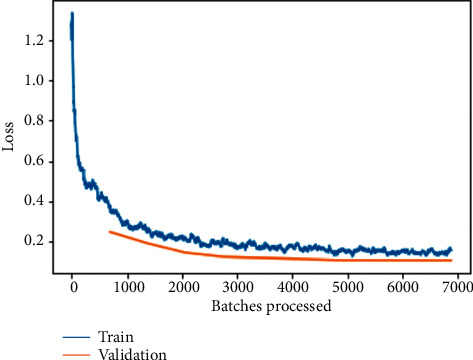
Training and validation loss on the basic VGG-16 model.

The training and validation loss before fine-tuning as shown in Figure 10 is a plot between loss and the number of batches processed for the VGG-16 model. In most deep-learning projects, the training and validation loss is usually visualized together on a graph. The purpose of this is to diagnose the model's performance and identify which aspects need tuning. The training loss is therefore frequently recognized half an epoch earlier than the validation loss, which is detected after each batch as a result of this. The validation loss provides the benefit of further gradient updates.

**Figure 11:**
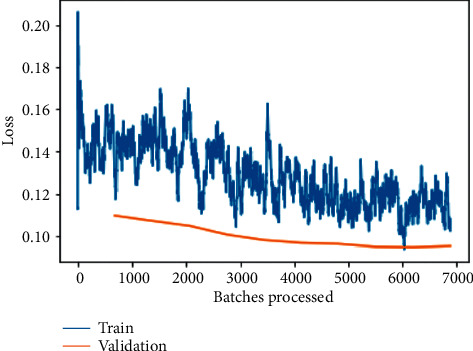
Training and validation loss on the fine-tuned VGG-16 model.

Here, it can be observed that loss is substantially being reduced exponentially as the number of batches is increased for processing; the same can be observed for training and validation loss on the fine-tuned VGG-16 model as shown in Figure 11 but with a little tilt of attaining constantly reduced values indicating an approximate straight line. The primary goal is to minimize the validity loss. It is almost always a good idea to overfit. There is nothing more important than making sure the risk of being disproved is as low as possible.

**Figure 12:**
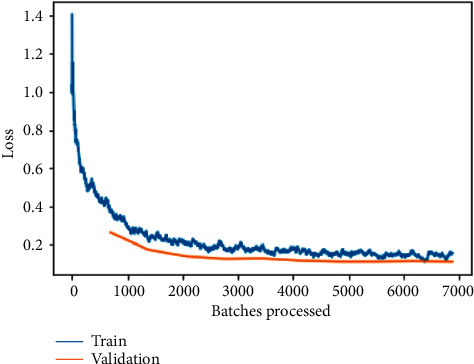
Training and validation loss on the basic VGG-19 model.

The training and validation loss before fine-tuning as shown in Figure 12 is a plot between loss and the number of batches processed for the VGG-19 model. While the model's training loss indicates how well it fits existing data, the validation loss reveals how well it fits brand-new data as input. Validation loss refers to the amount of data that is lost when it is divided into training, validation, and testing sets.

**Figure 13:**
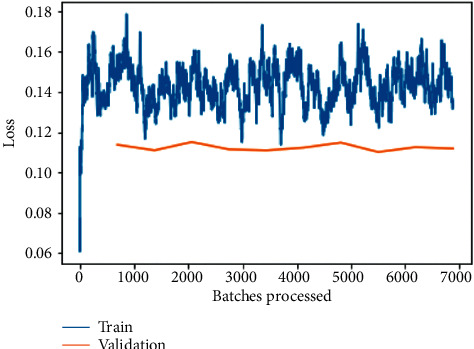
Training and validation loss on the fine-tuned VGG-19 model.

Here, it can be observed that loss is substantially being reduced exponentially as the number of batches is increased for processing; the same can be observed for training and validation loss on fine-tuned VGG-19 model as shown in Figure 13 but with a little tilt of attaining a sudden rise and then constantly reduced values indicating an approximate straight line. The most important goal is to minimize the validation loss to as little as possible. A little bit of overfitting is almost always a good idea. In the end, all that matters are that the loss of validation is as low as possible.

The training and validation losses per epoch for ResNet-50, ResNet-34, VGG-16, and VGG-19 are depicted in Figures 6–13 before and after fine-tuning for the four networks. As seen in Figures 7 and 9, the ResNet models are slightly overfitted after a few epochs; however, as shown in Figures 11 and 13, the VGG models are optimal. A more accurate way of putting it is that your model would be overfitting to the training data. Understanding the effects of overfitting is crucial to dealing with the problem. However, while high accuracy on the training set is often achievable, what you really want is to be able to design models that generalize well to a testing set (or data they have not encountered before).

Overfitting is the polar opposite of underfitting. Suboptimal fitting happens when the train data shows that there is still room for improvement to be made. For a variety of reasons, such as when the model is not powerful enough, when it is overregularized, or when it has not been trained for an adequate amount of time, this can occur. In this case, it shows that the network was unable to learn relevant patterns from the training data set.

## 6. Conclusion

To increase the performance of malaria diagnosis categorization in this study, we applied end-to-end deep-learning neural networks from start to finish. Deep learning helps computers find meaningful links in a large amount of data and make sense of unstructured data. Transfer learning, on the other hand, is a machine learning research problem that puts an emphasis on loading knowledge gained while trying to solve one problem and implementing it to a different but related problem. Based on our Fastai experience, we believe that using a layered API in deep learning can provide significant research benefits to the community. Compared to custom-built CNN models, it is, however, far more compatible with predefined architectures (for example, ResNet, Inception, and so on). Based on the simulation findings, it was proved that these deep-learning algorithms were capable of reaching extraordinarily high accuracy in pattern recognition. Based on our experimental findings, we conclude that the pretrained convolutional neural network model VGG-19 performs significantly better than ResNet-50, ResNet-34, and VGG-16 for the classification of blood smears. We employed transfer learning and fine-tuning to increase the performance of these pretrained models, and the results were promising. Their performance is influenced by the architecture, the training framework, and the volume of training data that they are given. In order to avoid information loss from images, the dropout approach was not employed in this study. We developed a web-based interface to make it easier for the end-user to use this model and categorize blood smear photos, which is now under development. This has the potential to reduce stress on medical field workers while simultaneously boosting the speed with which diagnoses are made. In the future, we want to concentrate on improving the performance of the CNN models by optimizing their architecture, which will result in a significant rise in the accuracy of malaria detection. Mobile devices and cloud-based implementation are also options for extending the end-user application's functionality.

## Figures and Tables

**Algorithm 1:**
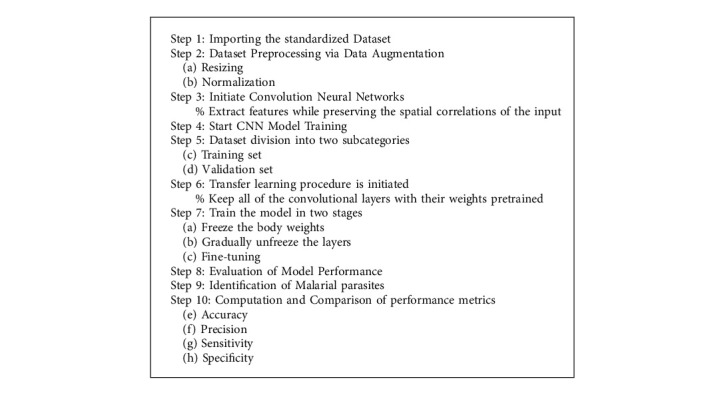


**Table 1:** Classification of the dataset.

Label	Training set	Validation set	Total
Parasitized	11,023	2,755	13,779
Uninfected	11,023	2,755	13,779

**Table 2:** Accuracies obtained before and after fine-tuning.

Model	Accuracy when fine-tuning is	
	Not applied	Applied
ResNet-50	0.957354	0.958265
ResNet-34	0.957358	0.963527
VGG-16	0.960624	0.960806
VGG-19	0.960987	0.972055

**Table 3:** Confusion matrix model.

		Predicted class	
		Class = yes	Class = no
Actual class	Class = yes	True positive (TP)	False negative (FN)
	Class = no	False positive (FP)	True negative (TN)

**Table 4:** Classification report of performance metrics.

Model	Accuracy	Sensitivity	Specificity	*F*1 score
ResNet-50	0.958265	0.966476	0.949759	1.919412
ResNet-34	0.963527	0.9671889	0.959734	1.917519
VGG-16	0.960806	0.970042	0.950868	1.926142
VGG-19	0.972055	0.979671	0.964166	1.939950

## Data Availability

The processed data are available upon request to the corresponding author.
